# Impact of Mixed-In Polyacrylic- and Phosphonate-Based Additives on Lime Mortar Microstructure

**DOI:** 10.3390/ma18143322

**Published:** 2025-07-15

**Authors:** Dulce Elizabeth Valdez Madrid, Encarnación Ruiz-Agudo, Sarah Bonilla-Correa, Nele De Belie, Veerle Cnudde

**Affiliations:** 1 Department of Geology, Ghent University, Krijgslaan 281/Building S8, B-9000 Ghent, Belgium; veerle.cnudde@ugent.be; 2 Department of Structural Engineering and Building Materials, Magnel-Vandepitte Laboratory, Ghent University, Zwijnaarde 60, B-9052 Ghent, Belgium; nele.debelie@ugent.be; 3 Department of Mineralogy and Petrology, University of Granada, Fuentenueva s/n, 18002 Granada, Spain; encaruiz@ugr.es (E.R.-A.); sbonilla@ugr.es (S.B.-C.); 4 Department of Earth Sciences, Utrecht University, Princetonlaan 8a, 3584 Utrecht, The Netherlands

**Keywords:** lime mortar, microstructure, porosity, poly(acrylic acid) sodium salt (PAA), aminotris(methylene phosphonic acid) (ATMP)

## Abstract

Aminotris(methylene phosphonic acid) (ATMP) and poly(acrylic acid) sodium salt (PAA) have shown favorable results in the treatment of porous building materials against weathering damage, showing promising potential as mixed-in additives during the production of lime-based mortars. This study investigates the impact of these additives on microstructure and mechanical properties. Additives were introduced in various concentrations to assess their influence on CaCO_3_ crystallization, porosity, strength, and carbonation behavior. Results revealed significant modifications in the morphology of CaCO_3_ precipitates, showing evidence of nanostructured CaCO_3_ aggregates and vaterite stabilization, thus indicating a non-classical crystallization pathway through the formation of amorphous CaCO_3_ phase(s), facilitated by organic occlusions. These nanostructural changes, resembling biomimetic calcitic precipitates enhanced mechanical performance by enabling plastic deformation and intergranular bridging. Increased porosity and pore connectivity facilitated CO_2_ diffusion towards the mortar matrix, contributing to strength development over time. However, high additive concentrations resulted in poor mechanical performance due to the excessive air entrainment capabilities of short-length polymers. Overall, this study demonstrates that the optimized dosages of ATMP and PAA can significantly enhance the durability and mechanical performance of lime-based mortars and suggests a promising alternative for the tailored manufacturing of highly compatible and durable materials for both the restoration of cultural heritage and modern sustainable construction.

## 1. Introduction

Lime mortars have been widely used both in historical and modern construction due to their workability, long-term durability, and compatibility with historical buildings [1,2]. Understanding their microstructural characteristics is crucial for optimizing their performance, particularly in heritage conservation and sustainable construction. For the purpose of material optimization, a well-established practice is the incorporation of organic and inorganic additives/admixtures in mortars and concrete. Evidence of the use of natural organic additives such as plant and herb extracts, oils, fats, and rice (amongst others) has been found in heritage building materials. These additives have been found to be responsible for the improvement in mechanical performance and durability of building materials [3,4,5]. For this reason, special interest is drawn to the effect of a wide variety of mixed-in additives on the workability, morphology, carbonation, hardening, and durability of concrete, mortars, and plasters for modern construction and restoration of cultural heritage [6,7]. At the same time, the use of additives can enhance the long-term resistance against environment- and human-driven decay, such as salt crystallization and freeze–thaw damage [8,9,10].

Recent studies have explored the use of polyacrylates and phosphonates such as poly(acrylic acid) sodium salt (PAA) and aminotris(methylene phosphonic acid) (ATMP) for the treatment of porous building materials to inhibit salt crystallization by modifying nucleation, crystal growth, and morphology in salt-exposed environments [11,12,13,14]. There is a growing need to explore the effects of these kinds of additives in lime mortars due to their potential of improving mortar stability and mitigating salt crystallization damage. PAA is a polyelectrolyte with carboxylic functional groups and has been shown to effectively modify crystal nucleation and growth dynamics due to its binding capacity [15,16]. Its adsorption onto mineral surfaces affects crystallization mechanisms and morphology, reducing the potential for salt-induced deterioration [13,14]. Research on mixed-in polymer-based additives has been conducted, related to their plasticizing and air-entraining effects and capacity to modify the rheology, workability, and mechanical properties of cementitious materials [8,17,18]. On the other hand, phosphonate-based inhibitors like ATMP have shown the effective inhibition of sodium sulfate crystallization, leading to reduced damage due to crystallization action [19].

Despite the promising results of PAA and ATMP as salt crystallization inhibitors for the treatment of porous materials, the role they play as mixed-in additives in lime mortar properties during hardening and their implications for mechanical performance are yet to be studied. Researchers investigated the impact of polymers and phosphonates such as PAA and ATMP during CaCO_3_ precipitation. For instance, Deng et al. [20] demonstrated the biomineralization of calcitic materials by the occlusion of organic additives as intracrystalline structural features in calcite. Furthermore, Rianasari et al. [21] and Diao et al. [22] explored the use of PAA for the template crystallization of CaCO_3_ simulating biominerals. Additionally, PAA has also been used for the controlled nucleation and synthesis of CaCO_3_ polymorphs by varying additive concentration and temperature [15,23,24,25,26], and has also been reported to influence the contact angle of calcium carbonate [27]. The conditions for the adsorption of phosphate-based compounds in different concentrations on specific calcite surfaces have also been studied, focused on their interaction in different concentrations with Ca^2+^ on specific calcite surfaces [28,29,30,31]. These studies highlight the significance of (in)organic inhibitors like ATMP and PAA in calcitic materials such as lime-based mortar formulations, particularly relevant for heritage conservation and restoration applications. Their ability to modify the mortar microstructure, performance, and their influence on salt crystallization offers promising strategies for the enhanced durability and mitigation of salt-induced deterioration in historical and modern construction materials.

This study aims to characterize the effects of both PAA and ATMP on lime mortar properties, including microstructural changes, mechanical strength evolution, and overall properties. By investigating different concentrations of PAA and ATMP, this research seeks to enhance lime mortar performance while also understanding the trade-offs between workability, porosity, strength, and carbonation rate. The findings will provide a comprehensive understanding of the role of PAA and ATMP in enhancing the properties of lime mortars, and insights into the development of more resilient and durable lime-based mortars for construction and restoration applications.

## 2. Materials and Methods

**Figure 1:**
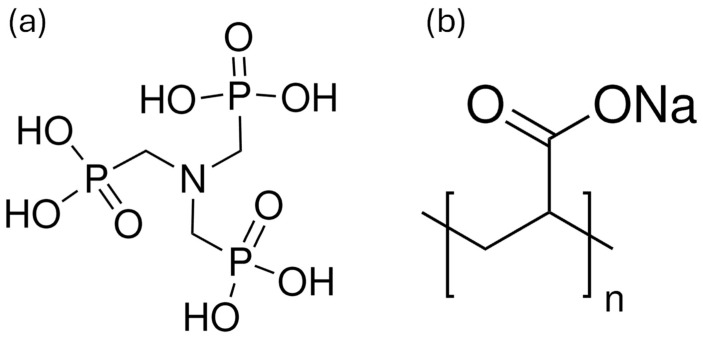
Chemical structures of (**a**) ATMP and (**b**) PAA molecules.

Lime mortars were made with a 1:3 binder-to-aggregate ratio (by volume) in compliance with EN 1015-11 [32], with the water-to-binder ratio (w/b) adjusted according to the flow table test [33] to obtain a flow of 165 ± 10 mm. CEN standard quartz sand (Normensand, Germany) was used for the aggregates and CL90-S hydrated lime for the binder (Lhoist, Belgium). A phosphonate (ATMP) (Figure 1a) and two polyacrylates (Sigma-Aldrich. Belgium) with different molecular weights (PAA5 with Mw 5100 and PAA2 with Mw 2100) (Figure 1b) were used during the mortar preparation by dissolving them in water in 100, 500, and 1000 ppm of the dry binder + aggregate mass, as specified in Table 1.

Prisms of 40 × 40 × 160 mm^3^ and cylinders of 25 mm diameter × 20 mm height were prepared based on the protocol in EN 1015-11 [32] for mortars exceeding 50% mass content of air–lime in the binder. After casting the mortar in two equal layers and compacting each one with 25 strokes of a tamper, two layers of cotton gauze and six layers of absorbent filter paper were laid above and below the mortar mixtures. A non-absorptive plate was then placed on top of the mold, followed by a 5 kg load for 3 h. Subsequently, the load, plate, cotton gauze, and filter paper layers were removed. The mortars were then cured in the mold for 5 days, then demolded and cured for 2 more days at 20 °C and 95% RH, followed by 21 or 83 days at 20 °C and 65% RH.

### 2.1. Microstructural and Mineralogical Characterization

#### 2.1.1. Nano-Scale Analysis

To examine morphological changes, polymorph stabilization, and additive integration in the carbonate matrix, nano-scale analyses were performed using Scanning Transmission Electron Microscopy (STEM) with a Thermo Fisher Scientific TALOS F200C G2 microscope (Scientific Instrumentation Center of the Health Campus of the University of Granada, Spain). Due to the limited availability of the equipment, observations were conducted on selected representative lime-based binders. Reference mortars without additives (REFs) and mortars containing 100 and 1000 ppm of ATMP, PAA5, and PAA2 were chosen to compare the effect of low and high amounts of additives after 90 days of curing. The mortar cylinders were split to obtain subsamples of the inner core. Silica grains were removed, and the binder was then homogenized using an agate mortar. Then, ethanol was added to facilitate dispersion, and a drop of the resulting suspension was deposited on a copper grid. A 70 μm objective aperture was used, and for selected area electron diffraction (SAED), a 10 μm aperture was applied to obtain a circular area approximately 0.2 μm in diameter. The STEM images were collected using a high-angle annular dark field (HAADF) detector.

#### 2.1.2. Microstructural Characterization

Field Emission Gun–Scanning Electron Microscopy coupled with Energy-Dispersive X-ray Spectroscopy (FEG-SEM-EDX) (TESCAN, Ghent, Belgium) was used to perform a microstructural assessment of binder–aggregate interactions of pure lime mortars (REFs) and mortars dosed with 100 and 1000 ppm of ATMP, PAA5, and PAA2. For this purpose, mortar cylinders (25 mm in diameter) of each composition were embedded in epoxy resin after 90 days of curing. Following a 24 h hardening period, the exposed surfaces were sequentially polished using silicon carbide grinding paper (grit 180–1000) and polycrystalline diamond suspension (15–3 µm) until a smooth mortar finish was achieved. The samples were then rinsed with demineralized water, dried overnight at 40 °C in a ventilated oven, and subsequently carbon-coated to enhance surface conductivity. Chemical analysis was performed on the ATMP-containing mortars to assess the homogeneity of the distribution of phosphonate-rich additives.

#### 2.1.3. Mineralogical Analysis

X-ray diffraction (XRD) analysis was performed on powdered samples to determine their mineralogy. Samples were ground in an agate mortar to a particle size below 50 µm using a PANalytical X’Pert PRO diffractometer (Granada, Spain) with CuKα radiation, λ = 1.5406 Å. Data were collected from 4 to 70 °2θ, with a time step of 10.16 [s] and a step size of 0.008 [°2θ]. The diffractograms were analyzed using the PANalytical HighScore Plus 3.0 software.

### 2.2. Porosity and Pore Size Distribution

The effect of increasing additive concentrations on the lime-based mortars’ pore size distribution and porosity was analyzed by mercury intrusion porosimetry (MIP) and X-ray micro-computed tomography (micro-CT). The MIP analysis was conducted on REFs and mortars dosed with 100, 500, and 1000 ppm of ATMP, PAA5, and PAA2 after 90 days of curing. For this purpose, 1–2 g of mortar fragments was obtained from the core of the mortar cylinders and left under vacuum for at least 7 days prior to analysis. A MicroActive AutoPore V 9600 (Micromeritics, Granada, Spain) equipment was used, with a Hg contact angle of 130° and a pressure from 0.007 to 420 MPa.

For micro-CT analysis, scans of mortar cylinders (25 mm diameter) were performed on REFs and mortars dosed with 100 and 1000 ppm of additives after 90 curing days to compare the effects of low versus high additive concentrations on their macroporosity. The scans were obtained using a CoreTOM scanner (TESCAN, Ghent, Belgium) (120 kV, 28 W, 600 ms exposure, 1 mm Al filter, 2142 projections). The Panthera software version 1.4.4. (TESCAN, Ghent, Belgium) was utilized for the 3D volume reconstruction, achieving a spatial resolution of 28 μm. The post-processing of the reconstructed volumes was conducted using the Avizo 3D 2022 software (ThermoFisher, Ghent, Belgium). A region of interest (ROI) was selected on every mortar cylinder comprising the same volume in all samples for comparison. Then, a *non-local means filter* module was applied to reduce noise, followed by the manual segmentation of the pores by an *interactive thresholding* module. For the pore volume quantification and visualization, the individual pores were isolated by a *separate objects* module and divided into 5 size fractions (<125 μm, 125–250 μm, 250–500 μm, 500–1000 μm, and >1000 μm) using a *sieving* module based on their equivalent diameter.

### 2.3. Natural Carbonation Rate and Mechanical Performance

Mortar prisms were used to determine the progress of the natural carbonation depth after 90 days of curing (horizontally), following EN 14630 [34]. Mortar prisms were split, perpendicular to their longitudinal axis, and a 1% phenolphthalein indicator solution in ethanol was used to spray the freshly broken faces (40 × 40 mm^2^). The color change from colorless to red-purple (due to its reaction with the higher pH of the uncarbonated binder) was recorded within 15 min of spraying. The carbonation depth, indicated by the non-colored areas, was measured to the nearest 0.1 mm, with at least 2 measurements per side taken per mortar mixture. The natural carbonation rate (NCR) was calculated by plotting the carbonation depth (mm) as a function of the square root of time (days) [35]. For the regression slope, carbonation depth at day 0 was assumed to be 0 mm.

Compressive and flexural strength were obtained on 40 × 40 × 160 mm^3^ mortar prisms according to EN 1015-11 [32] after 28 and 90 days of curing at standard conditions. A controls testing equipment (300/15 kN, Ghent, Belgium) was used, with a loading rate of 50 N/s at laboratory conditions (20 ± 5 °C, 60 ± 10% RH). Two prisms per mixture were tested. Additionally, to further assess the impact of additives on their long-term strength development, compressive strength tests were carried out on mortars after 480 days of curing.

## 3. Results and Discussion

### 3.1. Impact of Additives on Mortar Microstructure and Mineralogy

**Figure 2:**
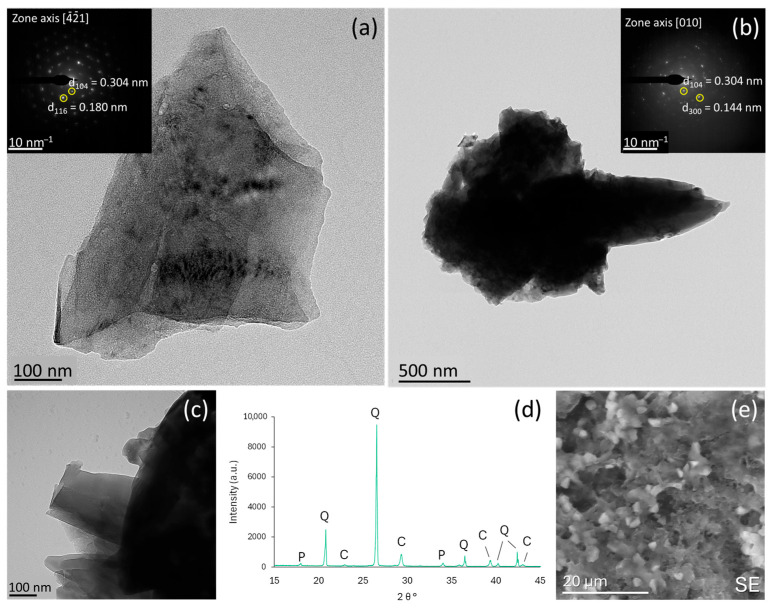
The TEM images of calcite crystals in reference mortars. (**a**) A rhombohedral crystal oriented along the [$\bar{4}\bar{2}1$] axis (see SAED pattern in the inset). (**b**) A scalenohedral crystal oriented along the [010] axis (SAED pattern in the inset). (**c**) The details of scalenohedral crystal showing smooth surfaces, with no rugosity apparent at the scale of observation. (**d**) The X-ray diffraction pattern of reference mortar in the presence of (P) portlandite, (C) calcite, and (Q) quartz. (**e**) The SEM image of rhombohedral calcite crystals in the reference mortar binder.

High-resolution transmission electron microscopy (HRTEM) analysis of the reference mortars reveals the presence of individual calcite crystals as well as aggregates of variable sizes (Figure 2). These crystals exhibit both rhombohedral (Figure 2a) and scalenohedral (Figure 2b) morphologies, characterized by smooth faces (Figure 2c), characteristic of the abiotic, inorganic origin of calcite. The crystals diffract as single crystals without angular spreading. Scalenohedral calcite has been frequently reported during the carbonation of lime mortars (e.g., Rodriguez-Navarro et al. [36], Cultrone et al. [37], and Cizer et al. [38]). The calcite cleavage rhombohedral (10$\bar{1}$4) faces correspond to F (flat) faces, parallel to two or more Periodic Boundary Conditions (PBCs) of the calcite structure, while the (21$\bar{3}$4) scalenohedral faces are S (stepped) faces, which include only one PBC. F faces are energetically more stable and typically dominate a crystal’s final growth form. In contrast, S faces are less stable and tend to grow continuously without becoming the primary habit-controlling crystal faces. Despite this, due to the polar nature of the scalenohedral faces (21$\bar{3}$4), these interact more strongly with the excess calcium ions compared to the non-polar, rhombohedral (10$\bar{1}$4) faces. This interaction ultimately contributes to the stabilization of the scalenohedral faces in the crystal structure, and their control on the morphology of the calcite crystals developed upon carbonation [38] (Figure 2e). This is evidenced by the presence of calcite in the X-ray diffraction pattern of the reference sample, along with portlandite (Ca(OH)_2_) and quartz from the aggregate fraction (Figure 2d).

**Figure 3:**
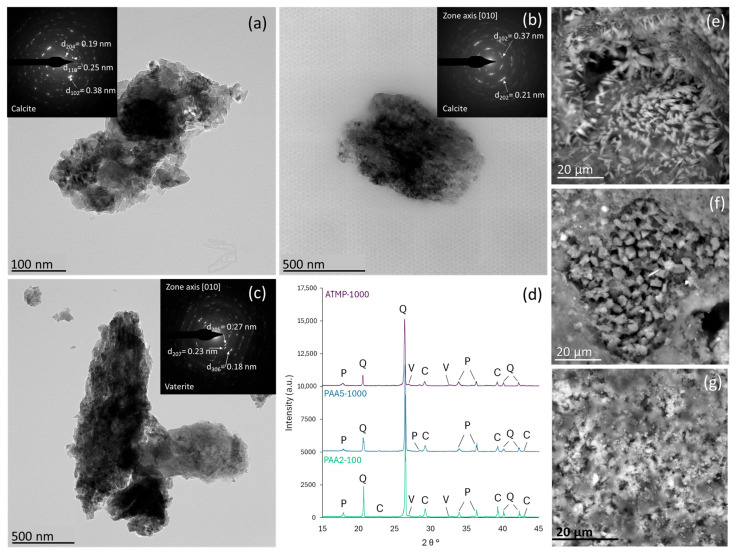
The TEM images of granular aggregates of CaCO_3_ in additive-bearing mortars, showing their mesocrystal nature with angular spreading between ~7° and 11° in the SAED patterns: (**a**) PAA2-100, (**b**) PAA5-1000, and (**c**) ATMP-1000 mortars. (**d**) X-ray diffraction patterns showing (P) portlandite, (Q) quartz, (V) vaterite, and (C) calcite. CaCO_3_ polymorphs observed in the SEM (BSE) images showed (**e**) scalenohedral calcite crystals in PAA2-100 mortar, (**f**) rhombohedral calcite crystals in PAA5-100 mortar, and (**g**) the presence of spherulitic vaterite structures in ATMP-1000 mortar.

In mortars doped with ATMP (1000 ppm) and PAA (100 and 1000 ppm), CaCO_3_ crystals exhibit a unique nanostructure composed of aggregated nanoparticles ranging from 30 to 100 nanometers in size (Figure 3). Their selected area electron diffraction (SAED) patterns reveal close to single-crystal characteristics with slightly arced diffraction spots (angular spread of approx. 7° to 11°). These observations are commonly seen in mesocrystals formed via an amorphous calcium carbonate (ACC) precursor through a non-classical oriented aggregation mechanism [39]. This was not so evident in the case of mortars doped with ATMP (100 ppm). d-spacings measured in the SAED patterns in additive-containing mortars coincided with those of calcite (Figure 3a,b), as well as of vaterite (Figure 3c), and were validated by their respective X-ray diffraction patterns (Figure 3d) and SEM observations (Figure 3e,f). This suggests that PAA and ATMP could help to stabilize crystalline precursors during lime carbonation. Previous studies have also reported the preservation of vaterite (and also aragonite) in completely carbonated lime mortars (e.g., M. Singh et al. [40] and Ouhenia et al. [23]). Although vaterite formation has been reported during the early stages of carbonation of mortars without organics, it rapidly transforms into calcite [41,42]. However, it is a relatively common observation that the presence of organic additives promotes the formation and stabilization of vaterite [43].

**Figure 4:**
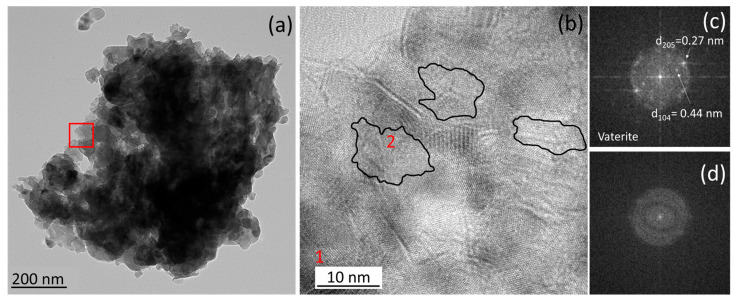
(**a**) The TEM image of a CaCO_3_ aggregate in PAA2-100 mortar. (**b**) The HRTEM image of the area marked with a red square in (**a**) showing lattice discontinuities (areas marked with black lines) due to amorphous inclusions. (**c**) FFT of area 1 (labeled in red in (**b**)) showing spots with d-spacings corresponding to vaterite. (**d**) FFT of area 2 (labeled in red in (**b**)); the absence of spots confirms the amorphous nature of this area.

Additionally, the HRTEM analysis of CaCO_3_ aggregates in PAA-containing mortars reveals low-contrast nanometer-scale areas in between individual CaCO_3_ nanodomains, i.e., vaterite in the example in Figure 4; see the Fast Fourier Transform (FFT) pattern of crystalline nanodomains in Figure 4c,d. These areas introduce discontinuities in lattice fringes and thus are suggested to correspond to an amorphous phase (see FFT of these areas in Figure 4d). Similar observations have been performed in historic mortars and replicas prepared with organics such as polysaccharide-rich plant extracts, and the amorphous areas in between nanocrystalline domains have been suggested to correspond to the organic additives (ATMP/PAA in our case) [3]. Overall, the presence of intercrystalline organic material within the newly formed CaCO_3_ in the binder appears to mimic the structure of calcium carbonate biominerals, such as sea urchin spines, which typically develop via an amorphous precursor phase and incorporate (bio)macromolecules across multiple scales [20].

**Figure 5:**
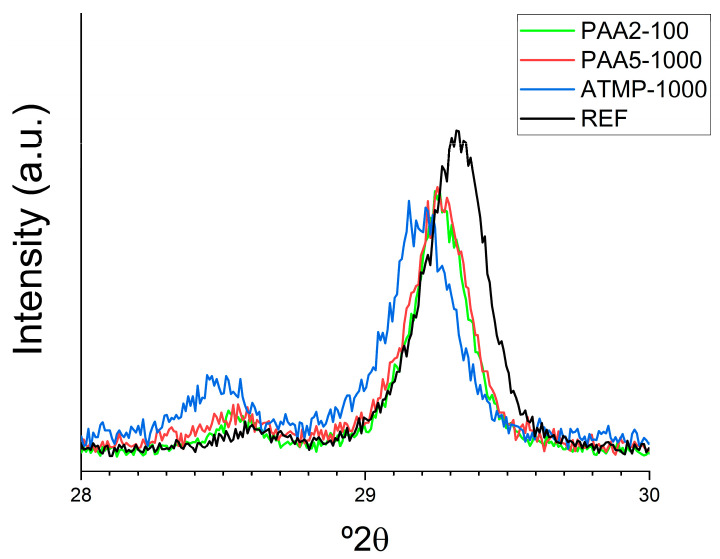
The XRD profiles of calcite 104 Bragg peaks of the reference mortars and mortars with additives showing peak broadening and shifting.

Broadening of the diffraction peaks was determined using the OriginPro software version 2016 for peak fitting to a Gaussian function (Figure 5). A marked peak broadening of the calcite 104 Bragg peak was observed in the X-ray diffraction pattern of ATMP-containing sample (Full Width at Half-Maximum, FWHM, = 0.254 vs. 0.246 for the reference sample). This broadening could be caused by various factors, including crystallite size reduction and an increased microstrain related to the distortion of the crystal lattice. In contrast, mortars containing PAA show the opposite behavior (FWHM = 0.237 and 0.221 for PAA5 and PAA2, respectively). Nevertheless, mortars including both PAA and ATMP clearly show a left shift in the calcite 104 Bragg peak position (i.e., an increase in d-spacing) when compared with reference mortars, particularly evident in the ATMP-containing sample. Such leftward shifts and peak broadening have been documented in biogenic and biomimetic calcite containing intracrystalline organic molecules, including calcitic cements in ancient mortars prepared using organics in their formulation [3]. Consistent with the HRTEM findings, the X-ray diffraction data validate the presence of organics as intracrystalline occlusions within the calcite cement of the studied organic-bearing mortars.

**Figure 6:**
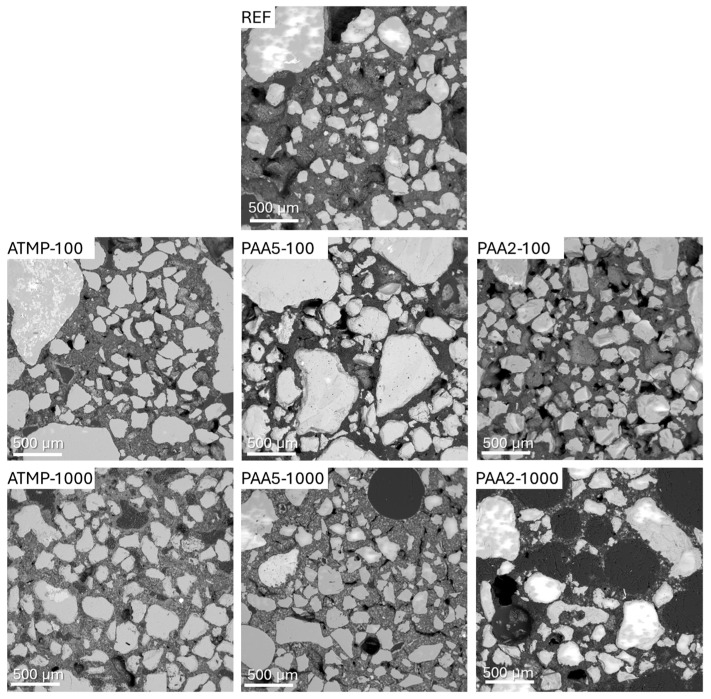
The FEG-SEM (BSE) images showing an overview of the microstructure of reference lime mortars and those dosed with 100 and 1000 ppm of additives.

Figure 6 shows the SEM micrographs with an overview of the mortar matrix and the interaction between the binder and the aggregates of the mortars with and without additives. Irregularly shaped pores were observed in most mortars, except for PAA5-1000 and PAA2-1000 mortars, which displayed bigger and evidently more round air voids between 100 and 500 µm. PAA5-1000 mortar also demonstrated the presence of microcracks along the binder and in some instances along the binder–aggregate interface (Figure 6). PAA molecules have a strong lubricating effect by binding water molecules to their carboxylic functional groups through the chemical combination of electrostatic forces, thus modifying the rheological behavior of the mortars [17]. While this binding capacity imparts a plasticizing effect, this particular affinity of PAA for Ca^2+^ ions and the available water molecules may have led to the excessive agglomeration of PAA5 in higher dosages (1000 ppm), potentially promoting the formation of fissures due to accelerated drying.

**Figure 7:**
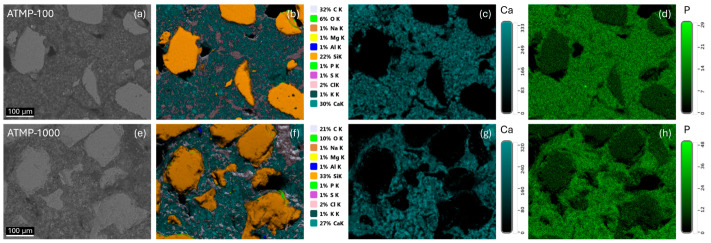
The representative FEG-SEM images of (**a**–**d**) ATMP-100 and (**e**–**h**) ATMP-1000 mortars. The EDX maps show homogeneous elemental distribution in the binder matrix (**b**,**f**), particularly Ca (**c**,**g**) from the CaCO_3_ binder and P (**d**,**h**) from the ATMP additive. The bars indicate the relative intensity of the elements in the region of interest.

The elemental analysis of mortars dosed with ATMP was conducted to assess the homogeneity of the additive distribution in the binder. Samples dosed with 100 and 1000 ppm of ATMP showed an overall homogeneous elemental distribution (Figure 7b,f). The relative intensity maps of calcium (Ca) (from the calcitic binder) and phosphorus (P) (from the ATMP additive) revealed a consistent distribution across the binder matrix on both ATMP-100 (Figure 7c,d) and ATMP-1000 mortars (Figure 7g,h). The EDX elemental quantification also revealed 50% increase in phosphorus (wt. %) in ATMP-1000 mortars compared to those containing 100 ppm, consistent with the higher additive dosage applied during mixing.

### 3.2. Porosity and Pore Size Distribution Results

**Figure 8:**
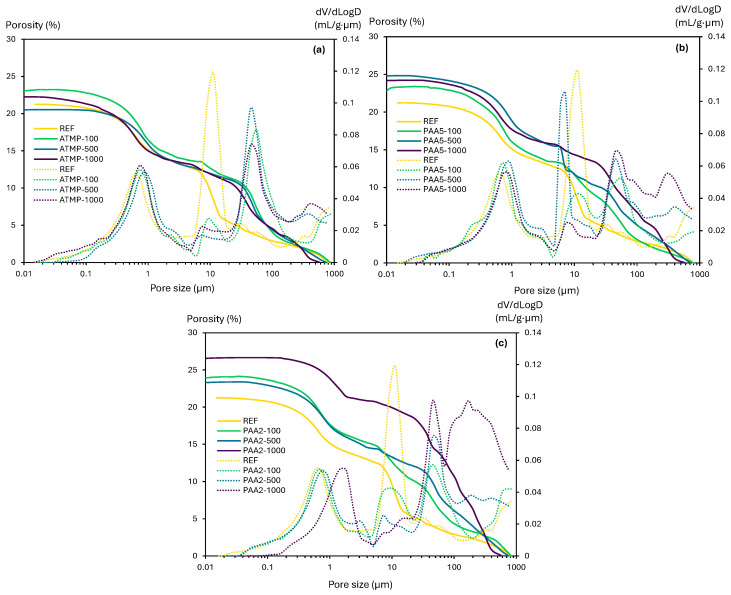
The open porosity and pore size distribution of lime mortars mixed with 100, 500, and 1000 ppm of (**a**) ATMP, (**b**) PAA5, and (**c**) PAA2 measured with MIP.

The porosity and pore size distribution of reference lime mortars and mortars containing additives were measured by MIP and micro-CT. First, the MIP analysis results (Figure 8) showed that all additive-dosed mortars exhibited an increase in total open porosity compared to the reference mortars, primarily due to the increased pore volume in the 20–1000 µm range. All ATMP-containing mortars showed a shift in pore size distribution from 10 to around 50 µm, along with an increase in pores at around 500 µm in ATMP-500 and ATMP-1000 mortars (Figure 8a). Additionally, peak shifts from 0.6 to ~2 µm were observed in all mortars containing ATMP, PAA5, and PAA2. PAA5-100, PAA5-1000, and all PAA2-containing mortars registered a reduction in pores around 10 µm, while the PAA5-500 mortars exhibited a shift from 10 µm to 7 µm (Figure 8b,c). All PAA5- and PAA2-bearing mortars showed an increase in pores at around 50 µm, and those with 500 and 1000 ppm of additive showed higher porosity at around 500 µm, consistent with the results of previous studies on lime mortars with mixed-in air entrainers [37]. Among all samples, PAA2-containing mortars exhibited the highest porosity increase due to the effect of the additive. Overall, mortars containing PAA5 and PAA2 resulted in a significant pore volume increase, primarily due to the increase in porosity above 50 µm, which is attributed to the air-entraining capabilities of polyacrylates such as PAA, especially at higher additive dosages [6,17,44].

**Figure 9:**
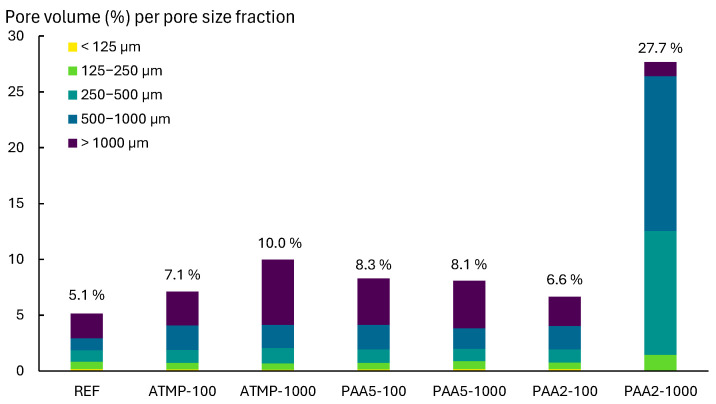
The pore volume (%) obtained from micro-CT image analysis of REFs and mortars with additives. The total pore volume (%) is divided into its pore size fractions. The % above the bars indicates the cumulative total porosity % per sample.

**Figure 10:**
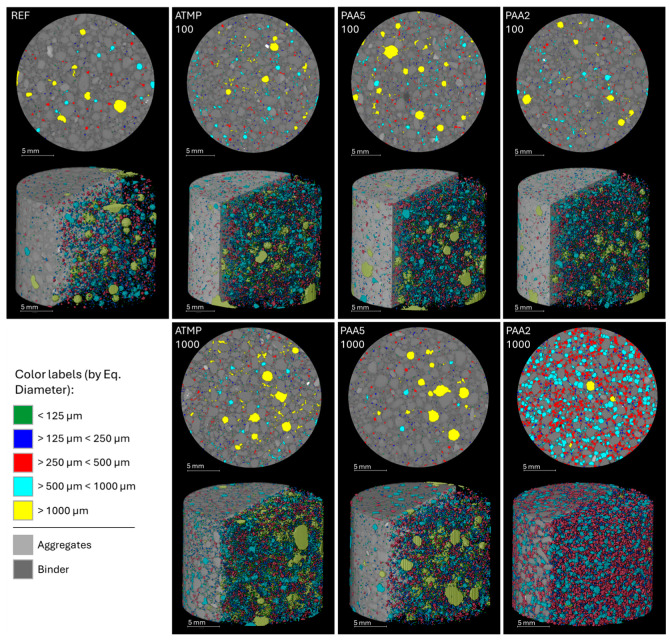
The 2D section and 3D volume visualization of pores divided by size fraction based on their equivalent diameter (in µm) obtained by micro-CT image analysis, showing the homogeneous distribution of pores across the mortar cylinders after 90 days of curing. Figure adapted from Valdez et al. [45].

The reference mortar and the mortars containing 100 and 1000 ppm of ATMP, PAA5, and PAA2 were selected for micro-CT image analysis to quantify changes in porosity above 56 µm (pore diameter), and to visualize the pore morphology and distribution across the mortar volumes. Results of the quantification displayed in Figure 9 illustrate that all mortars with additives exhibited an increase in total porosity volume compared to the reference. Additionally, 2D and 3D visualizations (Figure 10) evidenced homogeneous pore distribution on all mortars, together with more elongated and better interconnected pores in mortars with additives than the reference mortars. Mortars with ATMP and PAA2 dosed with 1000 ppm of additive showed a greater increase in porosity than those dosed with 100 ppm, whereas PAA5 mortars exhibited a limited decrease in total porosity when increasing the dosage from 100 to 1000 ppm. In ATMP-1000 mortars, the porosity increase was mainly attributed to the increase in pores larger than 1000 µm compared to ATMP-100. Similarly, PAA2-1000 mortars displayed a higher porosity than those with 100 ppm of additive. The air-entraining effect of PAA2 at higher concentrations (1000 ppm) is evident in Figure 10, where the increase in pores within the 250–1000 µm range is clearly visible, along with a more homogeneous distribution and greater interconnectivity between pores.

### 3.3. Natural Carbonation Rate Evolution

**Figure 11:**
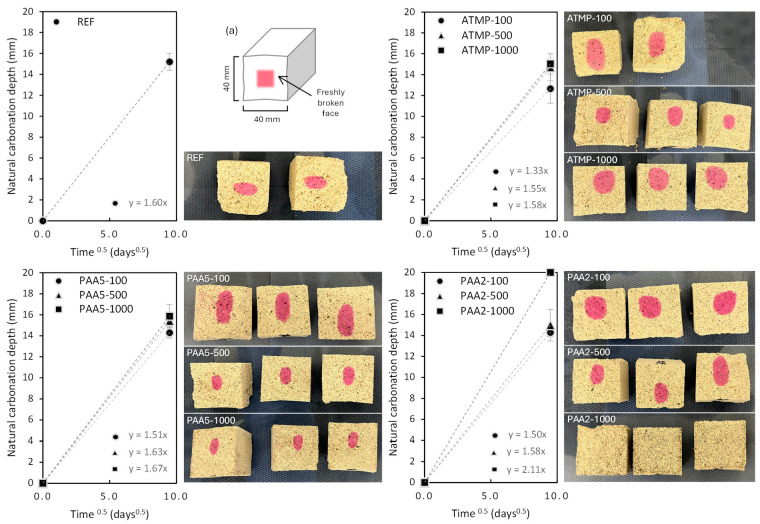
The natural carbonation rate (mm/days^0.5^) of lime mortars with and without additives determined by the phenolphthalein test method [34]. The error bars indicate the standard deviation of at least 4 measurements per mixture. (**a**) The diagram of the test setup, highlighting the measured surfaces. The images show the freshly broken surfaces after being sprayed with the phenolphthalein solution, showing in pink the uncarbonated area.

Results of the phenolphthalein test on lime mortars (Figure 11) showed that ATMP in all concentrations together with PAA5-100, PAA2-100, and PAA2-500 have a slower carbonation rate than the reference lime mortar. When using higher PAA5 dosage (500 and 1000 ppm), an acceleration in carbonation rate was observed. This could be related to the greater open porosity in mortars with higher additive dosage, promoting CO_2_ diffusion into the mortar matrix, thus accelerating the natural carbonation rate [39]. PAA2-1000 mortars did not show coloration after spraying with phenolphthalein, indicating the full carbonation of this mixture, possibly due to their high porosity above 50 µm and enhanced pore interconnectivity shown by micro-CT results.

### 3.4. Mechanical Performance

**Figure 12:**
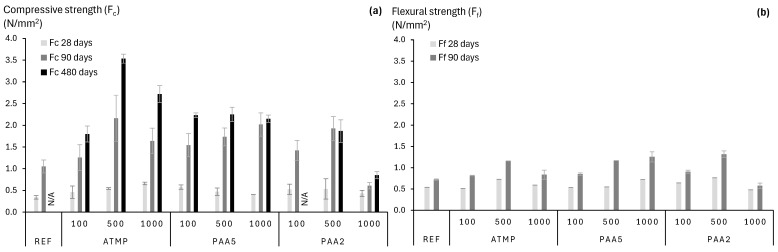
(**a**) The compressive (F_c_) and (**b**) flexural (F_f_) strength of mortars after 28, 90, or 480 days of curing at standard conditions. Error bars indicate standard deviation. REF and PAA2-100 samples after 480 days of curing were not tested; therefore, no values were obtained (N/A).

The evolution over time of the compressive and flexural strength of the reference mortar as well as mortars containing 100, 500, and 1000 ppm of additives is shown in Figure 12. After 28 days of curing, all mortars with additives, except for PAA2-1000, exhibited higher strength compared to the reference mortar without additives and continued to develop compressive strength up to 90 days. Most mortars with additives were further cured for 480 days, during which their compressive strength increased by 20–30% in certain cases (Figure 12a). REFs and PAA2-100 mortars were not tested at 480 days, given that at the time of testing, the loading rate and parameters selected for the compression testing appeared to be too high, leading to early failure with no additional specimens available for testing. Mortars containing ATMP and PAA2 reached their highest strength when using 500 ppm, while PAA5-containing mortars displayed comparable strength across all dosages. This strength evolution can be related to the continuous CO_2_ dissolution into the mortar matrix, promoting the advance of the carbonation front and increasing the degree of carbonation over time [39]. Furthermore, in Rodriguez-Navarro et al. [39], this continuous strengthening is further explained by calcite morphology evolution from scalenohedral to large rhombohedral crystals by continuous CO_2_ dissolution in the pore solution.

Strengthening of the materials was generally proportional to the additive dosage, except when a critical porosity threshold was exceeded. This was the case for PAA2-1000 mortars, which exhibited a significantly higher percentage of large air voids (>50 µm), similar to observations made by Riyazi et al. [44] and Cultrone et al. [37] when using polymer-based air entrainers in cement and lime mortars, respectively.

In previous studies by Rodriguez-Navarro et al. [3] and Deng et al. [20], it has been reported that organic occlusions (such as polysaccharide-rich plant extracts) in calcitic binders mimic biomineralization processes and can induce toughening, favoring plastic deformation and hindering fracture-induced failure [42]. In this work, organic occlusions occur through the presence of carboxylic and phosphonic functional groups in the polymer- and phosphonate-based additives, leading to strong bonding capacities with Ca^2+^ ions [10,12,29,30]. This has an enhanced effect in the presence of ATMP given its strong chemisorption onto the Ca^2+^ ions of the calcitic binder [31]. Consequently, the adsorption of these additives onto the carbonate-based binder promotes microstructure densification through the intercrystalline organic occlusions of PAA and ATMP. This occurs due to the introduction of discontinuities of amorphous phases in the lattice fringes, together with the stabilization of CaCO_3_ polymorphs, similar to the behavior of biogenic calcite [20].

### 3.5. Effect of Additives on Lime Mortars’ Microstructure and Performance

The addition of ATMP and PAA in different concentrations demonstrated a significant influence on the microstructural characteristics of lime-based mortars. These modifications were evident in the morphology of CaCO_3_ precipitates, as well as the porosity, strength, and carbonation rate of the hardened mortars. A summary of these modifications is displayed in Table 2.

Regardless of the additive type or concentration, SEM and micro-CT analyses revealed that all mortars consist of homogeneously distributed elemental compositions. The HRTEM results of the reference mortars evidenced the presence of rhombohedral and scalenohedral calcite, which is associated with the classical carbonation of Ca(OH)_2_ as the most stable polymorph of CaCO_3_. In contrast, mortars including ATMP (1000 ppm) and PAA (100–1000 ppm) facilitated the formation of nanostructured CaCO_3_ aggregates resembling mesocrystals, which are indicative of a non-classical crystallization pathway involving the initial stabilization of amorphous calcium carbonate (ACC) [39].

Moreover, HRTEM revealed the stabilization of vaterite (less stable CaCO_3_ polymorph) in mortars containing ATMP and PAA, together with amorphous intergranular regions in the PAA-dosed mortars, likely attributed to organic occlusions within the mineral matrix. Such structural features closely resemble biogenic calcite, wherein organics are occluded during crystal growth [20]. This type of micro- or nanostructure, with the presence of organic inclusions, may be responsible for the enhancement in the mortar’s mechanical performance.

It has been suggested that the presence of organics causes a toughening effect, since they facilitate plastic deformation, preventing the fracture-induced breakdown of the mortars [3]. However, the nanogranular structure of the mortars imprinted by the additives also contributes to such toughening effect, by providing intergranular bridging through the organics and enabling granular reorganization under stress. This effect was clearly observed by the enhanced strength of all mortars containing ATMP, PAA5, and PAA2 in all amounts (except PAA2-1000), regardless of their curing time. These characteristics potentially improve the material’s damage resistance, making it more resilient to mechanical stresses and thus to physical weathering processes such as salt crystallization pressures within pores [3]. Furthermore, this toughening and densification of the microstructure was more evident due to the increasing compressive strengths, reaching an optimal performance for ATMP- and PAA2-dosed mortars when using 500 ppm of additive, and 1000 ppm for PAA5 mortars, with continuous strength development over time. This was validated by one-way ANOVA tests assessing the effect of the additive concentrations on the compressive strength of the mortars at 90 days, with a significance level of 5%. The analysis revealed statistically significant differences among the mortar mixtures (*p* < 0.0001), with a large effect size (η^2^ = 0.77), indicating that additive type and concentration explain 77% of the variability in strength. Post hoc comparisons using Tukey’s HSD test showed that the ATMP-500 (*p*-value = 0.02%), PAA5-1000 (*p*-value = 0.1%), and PAA2-1500 (*p*-value = 0.5%) mixtures significantly improved compressive strength relative to the reference (REF). Conversely, PAA2-1000 showed a decrease in strength (*p*-value = 46.2%).

Simultaneously, the use of increasing additive dosages promoted the change in pore size distribution, shifting to bigger air voids (especially in the 20–1000 µm range), proportional to the amount of additive used. All mortars dosed with ATMP, PAA5, and PAA2 also displayed higher pore interconnectivity, which together with porosity volume promoted an increase in natural carbonation rate by favoring CO_2_ diffusion towards the mortar matrix [39]. This increased porosity did not compromise the mechanical properties of mortars with additives (except PAA2-1000 mortars), as they showed higher strength than the reference, and continued to increase up to 480 days after curing. This strength development over time can be related to the further advance of the carbonation front, leading to the densification of the microstructure. In addition, in previous research, this continuous strength gain has also been attributed to calcite morphology evolution towards large rhombohedral crystals due to continuous CO_2_ dissolution within the mortar matrix [39].

In contrast to PAA5-1000, PAA2-1000 mortars revealed a significant increase in pore volume and enhanced connectivity in the 250–1000 µm range. These characteristics are likely a direct result of the plasticizing effect and air-entraining capacity of PAA2, particularly in higher concentrations. PAA is characterized by its viscosity-enhancing capabilities, proportional to its molecular weight [17], explaining the higher viscosity of PAA5-1000 over PAA2-1000. This is clearly evident by the reduction in the water-to-binder ratio of PAA2-1000 needed to reach target fluence. While this enabled complete carbonation within 90 days of curing, it also resulted in the lowest compressive strength among all mortars tested. The high porosity and rapid carbonation appear to have disrupted the optimal microstructural development, leading to a mechanically weaker matrix.

## 4. Conclusions

This work assessed the effects on the microstructure and performance of lime-based mortars caused by the incorporation of aminotris (methylene phosphonic acid) (ATMP) and poly(acrylic acid) sodium salt in varying molecular weights (PAA5 and PAA2) and concentrations (100, 500, and 1000 ppm).

The presence of nanostructured aggregates and the stabilization of vaterite in all additive-dosed mortars indicated a shift from classical to non-classical crystallization pathways. These changes, confirmed by advanced imaging techniques and diffraction patterns, resulted in biogenic-like calcite structures with organic occlusions that enhanced mechanical performance. The presence of nanogranular features and intergranular organics promoted a toughening effect, improving mortar resistance to mechanical stresses, with optimal results observed at 500 ppm for ATMP and PAA2, and 1000 ppm for PAA5.

Despite increased porosity and pore interconnectivity, most additive-modified mortars demonstrated higher compressive strengths compared to the reference lime mortars. However, the PAA2-1000 mortar, while achieving rapid carbonation, exhibited excessive porosity and air entrainment due to its enhanced plasticizing effect, compromising its resulting strength. These findings suggest that, while organic additives can enhance durability and strength when using an optimal additive concentration, overly high doses may hinder optimal structural behavior.

Overall, the use of these organic additives offers promising pathways for the development of highly compatible materials for the restoration of cultural heritage, with the added benefit of enhanced mechanical performance and durability against freeze–thaw and salt crystallization damage. Future research on the long-term performance and compatibility with historical structures could provide additional insights into potential conservation strategies of these mixtures. Additionally, expanding the range of additives to include bio-based materials may lead to more sustainable and effective formulations, while suggesting new opportunities for the design of mortars inspired by biomimetic principles.

## Figures and Tables

**Table 1:** The composition of the mortar mixtures tested, including their chemical composition, weight-average molecular weight (Mw) of the additives used, and dosage of all components. The additive dosage (in ppm) was determined by the dry mass of binder + aggregates. All mortars were prepared with a 1:3 binder-to-aggregate ratio (by volume).

Sample ID	Additive Type	Additive (ppm)	Additive (g)	Lime (g)	Sand (g)	Water (g)	w/b Ratio (by Mass)
REF		-	-	113	1350	224	1.98
ATMP-100	N[CH_2_PO(OH)_2_]_3_ Mw: 299 g/mol	100	0.15	113	1350	224	1.98
ATMP-500		500	0.73	113	1350	224	1.98
ATMP-1000		1000	1.46	113	1350	224	1.98
PAA5-100	C_3_H_3_NaO_2_ Mw: 5100 g/mol	100	0.15	113	1350	224	1.98
PAA5-500		500	0.73	113	1350	224	1.98
PAA5-1000		1000	1.46	113	1350	210	1.86
PAA2-100	C_3_H_3_NaO_2_ Mw: 2100 g/mol	100	0.15	113	1350	224	1.98
PAA2-500		500	0.73	113	1350	210	1.86
PAA2-1000		1000	1.46	113	1350	196	1.73

**Table 2:** Summary of microstructural and mechanical effects of ATMP, PAA5, and PAA2 on lime mortars at various dosages.

Additive	Dosage (ppm)	Microstructural Features	Porosity ^1^	NCR ^2^	Mechanical Performance ^3^
REF	-	Rhombohedral and scalenohedral calcite + portlandite	Moderate	Baseline	Baseline
ATMP	100	Calcite + vaterite	Moderate	Significantly decreased	Improved
	500	Calcite + vaterite	Slight decrease	Significantly decreased	Significantly improved
	1000	Calcite + vaterite Mesocrystals via ACC precursor	High + predominance of >1000 µm	Increased	Improved
PAA5	100	Calcite + vaterite Mesocrystals via ACC precursor	Moderate	Decreased	Baseline
	500	Calcite + vaterite	Moderate + shift from 10 to 7 µm	Decreased	Baseline
	1000	Calcite + vaterite Mesocrystals via ACC precursor Increase in big spherical air voids and microcracks in the binder	High + predominance of >1000 µm	Increased	Baseline
PAA2	100	Calcite + vaterite Mesocrystals via ACC precursor Discontinuities due to organic occlusions	Moderate	Decreased	Improved
	500	Calcite + vaterite	Moderate increase	Decreased	Baseline
	1000	Calcite + vaterite Mesocrystals via ACC precursor Spherical air voids with high pore interconnectivity	Very high + predominance of 250–1000 µm	Significantly increased	Decreased

^1^ Based on mercury intrusion porosimetry (MIP) and micro-CT porosity volume quantification. ^2^ Based on a Tukey post hoc test of the natural carbonation rate (NCR) values obtained by EN 14630. ^3^ Based on a Tukey post hoc test using compressive strength (F_c_) values measured after 90 days of curing.

## Data Availability

The original contributions presented in this study are included in the article. Further inquiries can be directed to the corresponding author.

## Abbreviations

The following abbreviations are used in this manuscript:

ATMP	Aminotris(methylene phosphonic acid)
PAA	Poly(acrylic acid) sodium salt
STEM	Scanning Transmission Electron Microscopy
SAED	Selected area electron diffraction
FEG-SEM-EDX	Field Emission Gun–Scanning Electron Microscopy coupled with Energy-Dispersive X-ray Spectroscopy
XRD	X-ray diffraction
MIP	Mercury intrusion porosimetry
Micro-CT	X-ray micro-computed tomography
NCR	Natural carbonation rate
HRTEM	High-resolution transmission electron microscopy
PBCs	Periodic Boundary Conditions
FFT	Fast Fourier Transform
ACC	Amorphous calcium carbonate

## References

[1] Snow J., Torney C. (2014). Lime Mortars in Traditional Buildings.

[2] Faria P., Henriques F., Rato V. (2008). Comparative Evaluation of Lime Mortars for Architectural Conservation. J. Cult. Herit..

[3] Rodriguez-Navarro C., Monasterio-Guillot L., Burgos-Ruiz M., Ruiz-Agudo E., Elert K. (2023). Unveiling the Secret of Ancient Maya Masons: Biomimetic Lime Plasters with Plant Extracts. Sci. Adv..

[4] Thirumalini S., Ravi R., Rajesh M. (2018). Experimental Investigation on Physical and Mechanical Properties of Lime Mortar: Effect of Organic Addition. J. Cult. Herit..

[5] Zhang K., Sui Y., Wang L., Tie F., Yang F., Liu Y., Zhang Y. (2021). Effects of Sticky Rice Addition on the Properties of Lime-Tile Dust Mortars. Herit. Sci..

[6] Kontić A., Vasconcelos G., Briceño Melendez C., Azenha M., Sokolović N. (2023). Influence of Air Entrainers on the Properties of Hydrated Lime Mortars. Constr. Build. Mater..

[7] Nowak-Michta A. (2019). Impact Analysis of Air-Entraining and Superplasticizing Admixtures on Concrete Compressive Strength. Proceedings of the Procedia Structural Integrity.

[8] Silva B.A., Ferreira Pinto A.P., Gomes A., Candeias A. (2020). Suitability of Different Surfactants as Air-Entraining Admixtures for Lime Mortars. Constr. Build. Mater..

[9] Granneman S. (2019). Mitigating Salt Damage in Lime-Based Mortars by Built-In Crystallization Modifiers. Ph.D. Thesis.

[10] Granneman S.J.C., Lubelli B., Van Hees R.P.J. (2018). Characterization of Lime Mortar Additivated with Crystallization Modifiers. Int. J. Archit. Herit..

[11] Ruiz-Agudo E., Ibañez-Velasco A., Ruiz-Agudo C., Bonilla-Correa S., Elert K., Rodríguez-Navarro C. (2024). Damage of Porous Building Stone by Sodium Carbonate Crystallization and the Effect of Crystallization Modifiers. Constr. Build. Mater..

[12] Ruiz-Agudo E., Putnis C.V., Pel L., Rodriguez-Navarro C. (2013). Template-Assisted Crystallization of Sulfates onto Calcite: Implications for the Prevention of Salt Damage. Cryst. Growth Des..

[13] Andreotti S., Franzoni E., Ruiz-Agudo E., Scherer G.W., Fabbri P., Sassoni E., Rodriguez-Navarro C. (2019). New Polymer-Based Treatments for the Prevention of Damage by Salt Crystallization in Stone. Mater. Struct..

[14] Saleh M.M., Darwish S.S., Elzoghby M. (2022). The Effectiveness of Some Crystallization Inhibitors in Preventing Salt Damage to Limestone. J. Cryst. Growth.

[15] Huang S.C., Naka K., Chujo Y. (2008). Effect of Molecular Weights of Poly(Acrylic Acid) on Crystallization of Calcium Carbonate by the Delayed Addition Method. Polym. J..

[16] Rabizadeh T., Morgan D.J., Peacock C.L., Benning L.G. (2019). Effectiveness of Green Additives vs. Poly(Acrylic Acid) in Inhibiting Calcium Sulfate Dihydrate Crystallization. Ind. Eng. Chem. Res..

[17] Ma B., Peng Y., Tan H., Lv Z., Deng X. (2018). Effect of Polyacrylic Acid on Rheology of Cement Paste Plasticized by Polycarboxylate Superplasticizer. Materials.

[18] Paula A., Pinto F., Gomes A., Silva B.A., Silva B., Candeias A., Do Vale F. Role of Different Admixtures on the Properties of an Aerial Lime Mortar. Proceedings of the 3rd International Conference on Protection of Historical Constructions.

[19] Bracciale M.P., Sammut S., Cassar J.A., Santarelli M.L., Marrocchi A. (2020). Molecular Crystallization Inhibitors for Salt Damage Control in Porous Materials: An Overview. Molecules.

[20] Deng Z., Jia Z., Li L. (2022). Biomineralized Materials as Model Systems for Structural Composites: Intracrystalline Structural Features and Their Strengthening and Toughening Mechanisms. Adv. Sci..

[21] Rianasari I., Benyettou F., Sharma S.K., Blanton T., Kirmizialtin S., Jagannathan R. (2016). A Chemical Template for Synthesis of Molecular Sheets of Calcium Carbonate. Sci. Rep..

[22] Diao Y., Hu Q., Huang J., Guo X., Li P., Liu X., Bai J. (2024). Evaluation of Mechanical Properties and Potential Environmental Applications of Biomimetic Mineralized Composites. Constr. Build. Mater..

[23] Ouhenia S., Chateigner D., Belkhir M.A., Guilmeau E., Krauss C. (2008). Synthesis of Calcium Carbonate Polymorphs in the Presence of Polyacrylic Acid. J. Cryst. Growth.

[24] Xu A.W., Dong W.F., Antonietti M., Cölfen H. (2008). Polymorph Switching of Calcium Carbonate Crystals by Polymer-Controlled Crystallization. Adv. Funct. Mater..

[25] Wada N., Horiuchi N., Nakamura M., Nozaki K., Hiyama T., Nagai A., Yamashita K. (2015). Controlled Calcite Nucleation on Polarized Calcite Single Crystal Substrates in the Presence of Polyacrylic Acid. J. Cryst. Growth.

[26] Hu X., He P., Shi C. (2024). Carbonate Binders: Historic Developments and Perspectives. Cem. Concr. Res..

[27] Määttänen A., Ihalainen P., Bollström R., Wang S., Toivakka M., Peltonen J. (2010). Enhanced Surface Wetting of Pigment Coated Paper by UVC Irradiation. Ind. Eng. Chem. Res..

[28] Chen C., Lei W., Xia M., Wang F., Gong X. (2013). Molecular Modeling of Several Phosphonates onto the Stepped Calcite (011) Surface. Desalination.

[29] Sawada K., Abdel-Aal N., Sekino H., Satoh K. (2003). Adsorption of Inorganic Phosphates and Organic Polyphosphonate on Calcite. Dalton Trans..

[30] Kan A.T., Fu G., Tomson M.B. (2005). Adsorption and Precipitation of an Aminoalkylphosphonate onto Calcite. J. Colloid Interface Sci..

[31] Liu C., Zhu L., Fu W., Chi R., Li H., Yang S. (2022). Investigations of Amino Trimethylene Phosphonic Acid as a Green and Efficient Depressant for the Flotation Separation of Apatite from Calcite. Miner. Eng..

[32] (2019). Methods of Test for Mortar for Masonry—Part 11: Determination of Flexural and Compressive Strength of Hardened Mortar.

[33] (1999). Methods of Test for Mortar for Masonry—Part 3: Determination of Consistence of Fresh Mortar (by Flow Table).

[34] (2007). Products and Systems for the Protection and Repair of Concrete Structures—Test Methods—Determination of Carbonation Depth in Hardened Concrete by the Phenolphthalein Method.

[35] Van Den Heede P., De Schepper M., De Belie N. (2019). Accelerated and Natural Carbonation of Concrete with High Volumes of Fly Ash: Chemical, Mineralogical and Microstructural Effects. R. Soc. Open Sci..

[36] Rodriguez-Navarro C., Cazalla O., Elert K., Sebastian E. (2002). Liesegang Pattern Development in Carbonating Traditional Lime Mortars. Proc. R. Soc. A.

[37] Cultrone G., Sebastián E., Huertas M.O. (2005). Forced and Natural Carbonation of Lime-Based Mortars with and without Additives: Mineralogical and Textural Changes. Cem. Concr. Res..

[38] Cizer Ö., Rodriguez-Navarro C., Ruiz-Agudo E., Elsen J., Van Gemert D., Van Balen K. (2012). Phase and Morphology Evolution of Calcium Carbonate Precipitated by Carbonation of Hydrated Lime. J. Mater. Sci..

[39] Rodriguez-Navarro C., Ilić T., Ruiz-Agudo E., Elert K. (2023). Carbonation Mechanisms and Kinetics of Lime-Based Binders: An Overview. Cem. Concr. Res..

[40] Singh M., Vinodh Kumar S., Waghmare S.A., Sabale P.D. (2016). Aragonite-Vaterite-Calcite: Polymorphs of CaCO3 in 7th Century CE Lime Plasters of Alampur Group of Temples, India. Constr. Build. Mater..

[41] Silva B.A., Ferreira Pinto A.P., Gomes A., Candeias A. (2021). Effects of Natural and Accelerated Carbonation on the Properties of Lime-Based Materials. J. CO2 Util..

[42] You X., Hu X., Xiao Z., Saleh Bairq Z.A., Chen W., Shi C. (2023). Thermodynamic Modelling of CaCO_3_ Polymorphs during CO_2_ Sequestration by Cement Slurry with the Addition of MgCl_2_. J. Clean. Prod..

[43] Rodriguez-Navarro C., Jimenez-Lopez C., Rodriguez-Navarro A., Gonzalez-Muñoz M.T., Rodriguez-Gallego M. (2007). Bacterially Mediated Mineralization of Vaterite. Geochim. Cosmochim. Acta.

[44] Riyazi S., Kevern J.T., Mulheron M. (2017). Super Absorbent Polymers (SAPs) as Physical Air Entrainment in Cement Mortars. Constr. Build. Mater..

[45] Valdez Madrid D.E., Winardhi C.W., de Belie N., Cnudde V. (2024). Exploring Lime Mortar Microstructure: Investigating the Impact of Mixed-in Additives and their Role in Salt Efflorescence Inhibition. MATEC Web Conf..

